# Dose-Response Analysis Describes Particularly Rapid Repopulation of Non-Small Cell Lung Cancer during Concurrent Chemoradiotherapy

**DOI:** 10.3390/cancers14194869

**Published:** 2022-10-05

**Authors:** Huei-Tyng Huang, Michael G. Nix, Douglas H. Brand, David Cobben, Crispin T. Hiley, John D. Fenwick, Maria A. Hawkins

**Affiliations:** 1 Department of Medical Physics and Biomedical Engineering, University College London, London WC1E 6BT, UK; 2 Department of Medical Physics and Engineering, Leeds Cancer Centre, Leeds Teaching Hospitals NHS Foundation Trust, Leeds LS9 7TF, UK; 3 University College London Hospitals NHS Foundation Trust, London NW1 2BU, UK; 4 Clatterbridge Cancer Centre NHS Foundation Trust, Liverpool CH63 4JY, UK; 5 Department of Health Data Science, Institute of Population Health, University of Liverpool, Liverpool L69 3GF, UK; 6 Cancer Research UK Lung Cancer Centre of Excellence, University College London Cancer Institute, London WC1E 6AG, UK; 7 Department of Molecular and Clinical Cancer Medicine, Institute of Translational Medicine, University of Liverpool, Liverpool L69 7BE, UK

**Keywords:** NSCLC, radiotherapy, chemo-radiotherapy, tumour repopulation, radiation dose-response, radiotherapy schedules, data-modelling

## Abstract

**Simple Summary:**

Divergent results from trials of dose-escalation and acceleration suggest that optimal schedules have yet to be identified for radiotherapy of inoperable locally advanced non-small cell lung cancer. In this hypothesis-generating study radiation dose-response models were fitted to survival rates for 51 patient cohorts treated with schedules of varying total radiation dose, dose-per-fraction and duration. The best fit described repopulation running at 1.47 Gy/day for concurrent chemoradiotherapy and 0.30 Gy/day for radiotherapy alone and sequential chemoradiotherapy. The overall fitted tumour *α*/*β* ratio was 3.0 Gy. These findings imply that moderate hypofractionation of chemoradiation, within normal tissue toxicity limits, should be efficacious.

**Abstract:**

(1) Purpose: We analysed overall survival (OS) rates following radiotherapy (RT) and chemo-RT of locally-advanced non-small cell lung cancer (LA-NSCLC) to investigate whether tumour repopulation varies with treatment-type, and to further characterise the low *α*/*β* ratio found in a previous study. (2) Materials and methods: Our dataset comprised 2-year OS rates for 4866 NSCLC patients (90.5% stage IIIA/B) belonging to 51 cohorts treated with definitive RT, sequential chemo-RT (sCRT) or concurrent chemo-RT (cCRT) given in doses-per-fraction ≤3 Gy over 16–60 days. Progressively more detailed dose-response models were fitted, beginning with a probit model, adding chemotherapy effects and survival-limiting toxicity, and allowing tumour repopulation and *α*/*β* to vary with treatment-type and stage. Models were fitted using the maximum-likelihood technique, then assessed via the Akaike information criterion and cross-validation. (3) Results: The most detailed model performed best, with repopulation offsetting 1.47 Gy/day (95% confidence interval, CI: 0.36, 2.57 Gy/day) for cCRT but only 0.30 Gy/day (95% CI: 0.18, 0.47 Gy/day) for RT/sCRT. The overall fitted tumour *α*/*β* ratio was 3.0 Gy (95% CI: 1.6, 5.6 Gy). (4) Conclusion: The fitted repopulation rates indicate that cCRT schedule durations should be shortened to the minimum in which prescribed doses can be tolerated. The low *α*/*β* ratio suggests hypofractionation should be efficacious.

## 1. Introduction

Over 80% of all lung cancers are non-small cell (NSCLC) [1] and more than 60% of patients with NSCLC receive radiotherapy (RT) at some point [2]. For early-stage patients unfit for surgery, stereotactic body radiotherapy is now an accepted standard of care [3]. For patients with inoperable locally advanced (LA-) NSCLC the best chance of survival is offered by concurrent chemo-RT (cCRT), given as 60–66 Gy in daily 2 Gy fractions with 2–4 chemotherapy cycles [4,5]. However, many patients are insufficiently fit for cCRT and instead receive RT alone or sequential chemo-RT (sCRT).

It remains challenging to identify optimal RT schedules for LA-NSCLC. Compared to conventional fractionation, a 12-day accelerated hyperfractionated course of RT alone achieved improved 2-year overall survival (OS) (29% vs. 20%, *p =* 0.004) but tumours nevertheless recurred in 47% of patients receiving the accelerated treatment [6]. Meta-analyses found that survival was improved by combined chemo-RT, and that cCRT achieved an absolute survival benefit of roughly 5% compared to sCRT [7,8]. Data from some early-phase clinical trials of radiation dose-escalation showed improved OS [9] and acceptable toxicity [10], and a meta-analysis showed survival gains for escalation of RT alone and sCRT [11]. For cCRT, however, a survival gain was not seen and the *RTOG-0617* phase III trial reported a median survival of 20.3 months for 74 Gy in 2 Gy fractions compared to 28.7 months for the baseline 60 Gy treatment [12], a survival reduction of 8.4 months in the high-dose arm (*p =* 0.004). These findings are not coherently explained by standard in-silico models, and improved models are needed to guide the optimization of radiation treatments.

Over the last decade this challenge has begun to be addressed. In a 2011 analysis of results from 24 published trials, local disease-free survival was found to rise with increasing tumour equivalent dose in 2 Gy fractions (EQD_2_) [13]. In the study, EQD_2_ values were calculated for each schedule using the linear-quadratic model with standard values of the tumour *α/β*, *λ* and *T_k_* parameters. These radiobiological indices respectively describe the extent to which tumour cell-killing depends on the schedule dose-per-fraction as well as total radiation dose, the dose-per-day lost to accelerated repopulation during RT treatment, and the kick-off time after the start of RT at which repopulation begins. In a later analysis of 2-year OS carried out by Nix et al. for an extended dataset [14], an improved description of survival data was achieved using a model that included fitted tumour *α/β* and repopulation parameters, a term representing possible reductions in survival due to toxicity effects, and factors accounting for control of metastases by chemotherapy and radiosensitisation of the primary tumour during cCRT. This model fit included an unexpectedly low tumour *α/β* value of 4.0 Gy and described OS rates first rising with increasing EQD_2_ before falling again. In a plot of survival levels calculated from the model for cCRT treatments given in increasing numbers of 2 Gy fractions over 40 days, survival for stage IIIA patients started to fall as doses increased beyond 68 Gy. This was a lower turnover point than for RT alone or sCRT, for which modelled survival continued to rise through to 80 Gy.

In the present study we determine whether further generalisations of Nix’s model improve the description of the survival data, potentially better informing the choice of dose, dose-per-fraction and treatment duration for optimising chemoradiation schedules. First, we explore whether tumour accelerated repopulation varies with treatment-type (cCRT vs. RT/sCRT) in accordance with a recent proposal that cCRT might suppress repopulation, negating the effect of treatment acceleration [15]. Second, we investigate the origin of the low *α/β* ratio, studying whether it is driven by a particular disease stage or treatment-type. And finally, we allow the survival-limiting term to vary with stage, since larger volumes of normal tissues are irradiated to high dose-levels during treatment of higher stage disease. By building a baseline dataset and dose-response model for chemo-RT, we also aim to create a platform for future work characterising the additional effects on survival of combined immune-chemo-RT treatments.

## 2. Materials and Methods

### 2.1. Data

The dataset analysed was derived from that of Nix et al. [14], who identified data sources from *PubMed*, *Google Scholar* and *ScienceDirect* searches for ‘NSCLC radiotherapy dose-escalation’ and from further citation-following. The original dataset included studies published from 1995–2016 which detailed 2-year OS for cohorts of >20 patients treated using doses ≤4.0 Gy per fraction, weighting the data towards higher stage disease since early-stage patients often receive hypofractionated treatments. The current dataset excludes studies published before 2000 to better focus on the conformal and intensity-modulated RT era. Cohorts comprising ≥60% stage I-II patients (American Joint Committee on Cancer stage definition versions 4–7) were also excluded, as were cohorts given >3.0 Gy per fraction in order to base the *α/β* analysis on a conventional range of fractionation. For studies in which lung heterogeneity corrections had not been made, we raised tabulated prescribed doses and doses-per-fraction by 5% above published values [16].

### 2.2. Dose-Response Models

**Figure 1:**
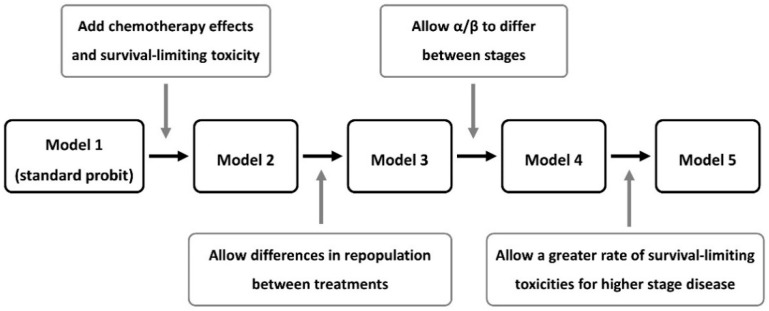
Flow-chart of the overall survival (OS) models investigated.

Initially a probit-type dose-response model was fitted to the data, then the model of Nix et al. [14] was fitted, followed by three incremental generalisations. These five principal models are illustrated in Figure 1 and detailed below.

#### 2.2.1. Model 1: Standard Probit Dose-Response

Tumour EQD_2_ was calculated for each cohort and corrected for treatment duration via:

(1) $$EQD_{2,tum-RT}=D\times \left(\alpha /\beta +d\right)\;/\left(\alpha /\beta +2\right)-\lambda \times \mathrm{Max}\left[T-T_{k},0\right]$$

where *D* is the total prescribed dose, *d* the dose-per-fraction and *T* the duration of RT used to treat the cohort. The quantities *α/β*, *λ* and *T_k_* are fitted radiobiological parameters that describe the tumour fractionation sensitivity, dose-per-day lost to repopulation and the repopulation start time [13]. Then EQD_2_ was linked to 2-year OS probability for disease stage *S_i_* through the cumulative normal (probit) sigmoid-shaped link-function *ϕ*:

(2) $$OS_{2-year-S_{i}}\left(EQD_{2,tum-RT}\right)=\phi \left[\frac{EQD_{2,tum-RT}-EQD_{2,tum50}\left(S_{i}\right)}{m\times EQD_{2,tum50}\left(S_{i}\right)\;}\right]\times 100\%$$

where the fitted parameters *m* and *EQD*_2*,tum*50_(*S_i_*) describe the slope of the tumour control probability dose-response curve and the prescribed EQD_2_ required to achieve 50% tumour control for stage *S_i_*, respectively. Finally, survival for the whole cohort was calculated as the weighted average:

(3) $$OS_{2-year-probit}\left(EQD_{2,tum-RT}\right)=\overset{}{\underset{i}{\sum }}f_{i}\times OS_{2-year-S_{i}}\left(EQD_{2,tum-RT}\right)$$

in which *f_i_* is the fraction of patients in the cohort with stage *i* NSCLC.

#### 2.2.2. Model 2: Nix Model including Chemotherapy, Survival-Limiting Toxicity and Date Effects

Tumour chemo-radiosensitisation was accounted for by a factor *RS* fixed at 1 for RT alone and sCRT but fitted to values >1 for cCRT.

(4) $$EQD_{2,tum-CRT}=RS\times D\times \left(\alpha /\beta +d\right)\;/\left(\alpha /\beta +2\right)-\lambda \times \mathrm{Max}\left[T-T_{k},0\right]$$

The systemic effect of chemotherapy was added to the model along with the effects of survival-limiting toxicity and publication year through:

(5) $$OS_{2-year-Nix}\left(EQD_{2,tum-CRT}\right)=\;OS_{2-year-probit}\left(EQD_{2,tum-CRT}\right)\times OS_{max}\times \left(1-SLT\right)\times \left(1-R\times Y\right)$$

where *OS_max_* was fixed at 85% for cohorts receiving RT alone, corresponding to a 15% rate of distant failure seen in patients not undergoing chemotherapy [14,17], but was fitted to a common value >85% for cohorts treated using sCRT or cCRT.

The *SLT* term in Equation (5) represents the probability of possible survival-limiting toxicities occurring at high radiation doses and is calculated as:

(6) $$SLT=\phi \left[\frac{EQD_{2,NT}-EQD_{2,NT50}\;}{m_{NT}\times EQD_{2,NT50}}\right]$$

in which *EQD*_2*,NT*_ is obtained from the prescribed dose using Equation (1) with no repopulation and the generic *α/β* ratio of 3 Gy for normal tissues, and the fitted parameters *m_NT_* and *EQD*_2*,NT*50_ define the dose-response gradient and EQD_2_ at which such toxicities would occur in 50% of patients. The number of years a study was published before 2016 is denoted by *Y* in Equation (5), and the fitted parameter *R* accounts for any increase in survival with publication year due to treatment improvements over time unrelated to changes in dose or chemotherapy schedules.

#### 2.2.3. Model 3: Adding Treatment-Dependent Repopulation

The fitted *λ* and *T_k_* repopulation parameters of Equation (1) were allowed to take different fitted values for cCRT vs. RT alone or sCRT.

#### 2.2.4. Model 4: Adding Stage-Dependent Fractionation Dependence

The fitted tumour *α/β* ratio in Equation (1) was allowed to take different values for stage I/II vs. stage IIIA vs. IIIB NSCLC.

#### 2.2.5. Model 5: Adding Stage-Dependent Toxicity

The survival-limiting toxicity term was varied with stage *S_i_* disease through:

(7) $$SLT\left(S_{i}\right)=F_{SLT-i}\times \phi \left[\frac{EQD_{2,NT}-EQD_{2,NT50}\;}{m_{NT}\times EQD_{2,NT50}}\right]$$

where the fitted *F_SLT-i_* (≤1) values conceptually represent the fraction of stage *S_i_* patients in whom high, prescription-level doses overlap the critical normal tissues in which survival-limiting toxicities might originate.

### 2.3. Statistics

Data analyses were performed using *RStudio* software (version 1.2.5033). Model fitting was carried out via maximum-likelihood estimation using the *bbmle* package [18]. Dose-response curves were plotted using *ggplot2* [19]. Confidence intervals (CI) on fitted parameters were determined using the profile-likelihood method, which refits the model with the parameter of interest fixed to a series of values, and identifies the CI for the parameter as the range over which twice the negative log-likelihood increases by less than $\chi_{\left(1,0.95\right)}^{2}$ = 3.84 from the best-fit [20].

The Akaike information criterion (AIC) was used to assess which model described the data best, checking results via 10-fold cross-validation and using the likelihood-ratio test to determine significances of fit improvements made by adding new parameters to models [21]. The AIC score represents the scale of differences between datapoints, and a model fit to them, with a penalty term equal to twice the number of fitted model parameters added, and the model with the lowest AIC score is considered best. Thus, the AIC balances the need to describe the data well against the tendency for overly complex models to over-fit the data, describing the statistical noise as well as the underlying information. For the best model, the asymptotic correlation matrix of fitted model parameters was plotted using *corrplot* [22].

Linear fits to cohort data in scatter plots were determined by minimising sums of squared errors weighted by patient numbers, and significances of gradients were assessed using the F-test. Significances of differences in treatment characteristics between sets of cohorts were assessed using the Wilcoxon rank-sum test. All reported significance values are two-sided.

## 3. Results

### 3.1. Data

The dataset comprised 4866 patients with stage I (5.1%), II (4.4%), IIIA (46.6%) or IIIB (43.9%) NSCLC. The patients belonged to 51 cohorts treated in 33 studies published between 2000 and 2016. Tumour histology data was available for 39 cohorts in which 43%, 30% and 28% of patients had squamous cell carcinoma, adenocarcinoma and other NSCLC histologies (Table 1).

Of the 51 cohorts, 10 were treated using RT alone, 26 using sCRT (12 neo-adjuvant, 1 adjuvant, 13 mixed or not clearly indicated) and 15 using cCRT. Schedules were borderline significantly shorter for RT alone than for sCRT (*p =* 0.026) or cCRT (*p =* 0.055), with mean durations of 35, 42 and 43 days for the three treatment-types. Conformal RT techniques were used to treat patients in 40 cohorts, while intensity-modulated RT was used for six cohorts and unmodulated rectangular fields for five.

**Figure 2:**
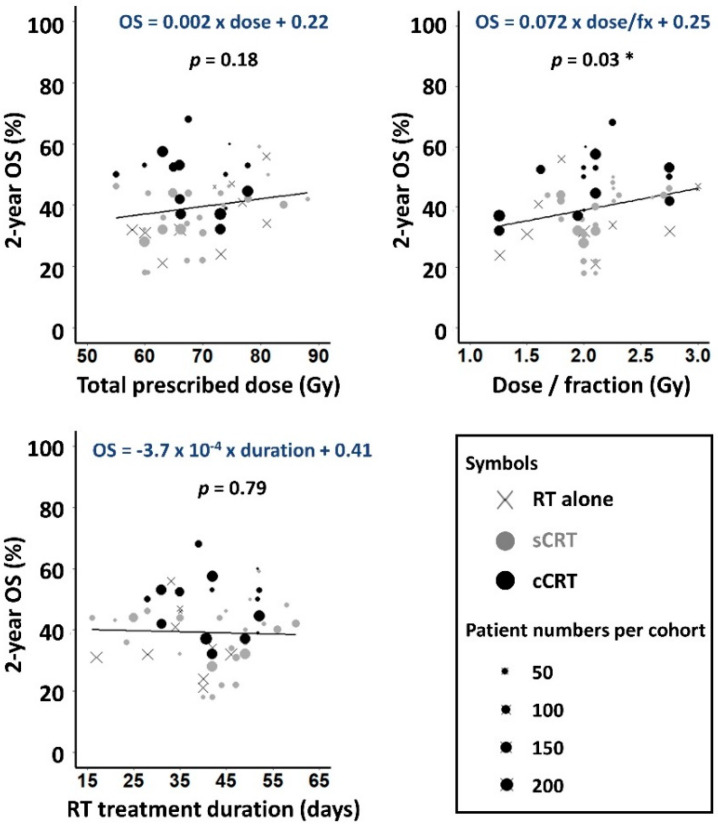
Observed 2-year OS plotted against prescribed dose, dose-per-fraction and RT duration for the 51 cohorts. Weighted least squares linear fits to the data are shown, with gradients significantly different to zero asterisked (*p* < 0.05).

Reported 2-year OS ranged from 18% to 68%. On univariable linear regression analysis OS increased insignificantly with physical dose, by 0.2% absolute per Gy (Figure 2), lower than dose-response gradients of 1–3%/Gy reported for various tumour-types [23]. OS rose significantly with increasing dose-per-fraction, at a rate of 7% per Gy/fraction (*p =* 0.028) but did not vary significantly with treatment duration.

### 3.2. Model Fits

Fits of Models 1, 2 and 5 to the survival data are presented in Table 2, and fits of all five models are described in Tables S1 and S2. The data were described significantly better by Model 2, which accounted for survival-limiting toxicity and the radiosensitising and systemic effects of chemotherapy, than by the standard probit Model 1 (likelihood-ratio test, *p <* 10^−20^). Model 2 also had better AIC and cross-validation scores, consistent with our earlier analysis of Nix’s dataset [14].

**Figure 3:**
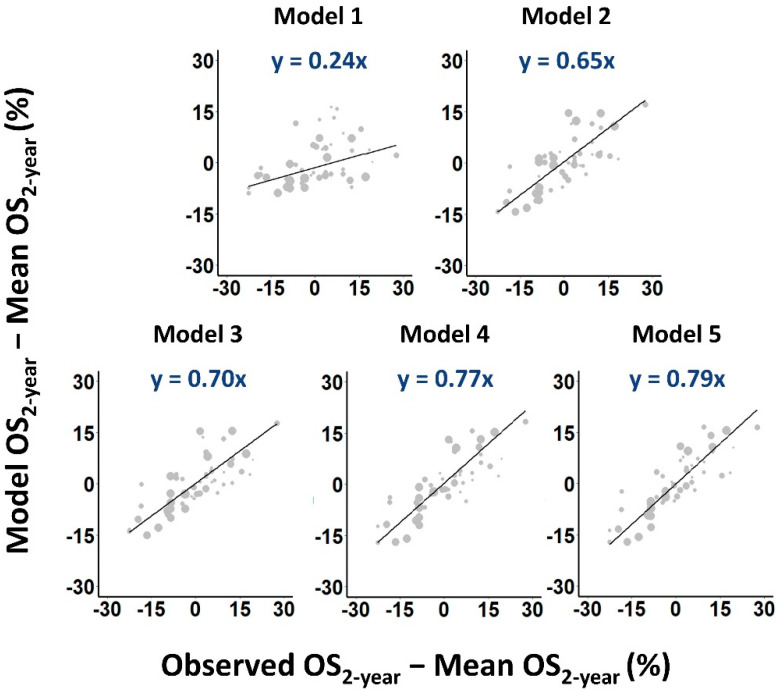
Calibration plots of predicted versus observed OS_2-year_ rates. Larger symbols are used to indicate cohorts with more patients. Weighted least squares linear fits to the data are shown as solid lines.

The more general Model 5, which included repopulation parameter values that depended on treatment type, and *α/β* and survival-limiting toxicity parameter values that depended on disease stage, fitted the data significantly better again (*p <* 10^−4^) and performed best according to AIC and cross-validation scores (Table 2). In fact, each incremental generalization from Model 2 to Model 5 led to a significant improvement in the quality of the fit (Table S2), reducing dispersion and steepening regression gradients in calibration plots (Figure 3).

In the fit of Model 1, accelerated repopulation ran at 0.64 Gy EQD_2_-per-day (95% CI: 0.30, 0.99 Gy/day) beginning at day 33 (95% CI: 18, 39) of RT. In Model 5 the fitted repopulation rate was 0.30 Gy/day (95% CI: 0.18, 0.47 Gy/day) for RT alone and sCRT, slower than in the probit model though beginning earlier at day 17 (95% CI: 16, 32). For cCRT, however, repopulation in Model 5 ran at 1.47 Gy/day (95% CI: 0.36, 2.57 Gy/day) starting at day 24 (95% CI: 16, 47), suggesting that tumour repopulation begins later for cCRT but at an augmented rate.

**Figure 4:**
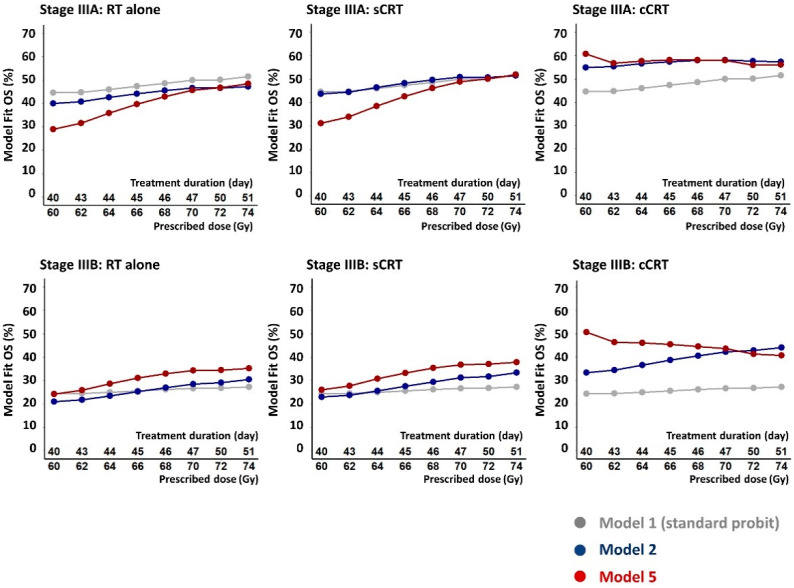
Calculated 2-year OS rates for stages IIIA and IIIB NSCLC patients treated with RT alone, sCRT and cCRT, plotted for a range of possible schedules giving 60–74 Gy in 2 Gy fractions five days per week over 40 to 51 days. The OS rates were calculated from fits of the standard probit model (Model 1), the Nix model (Model 2) and the full model (Model 5) which describes the fitted data best according to the AIC and cross-validation measures.

Figure 4 illustrates implications of the model fits for treatment design. 2-year OS rates calculated from the fit of Model 5, which best describes the data, are plotted for a range of possible schedules delivering doses of 60–74 Gy in 2 Gy fractions given five days-per-week. For RT alone and sCRT, calculated survival rates rise progressively with increasing dose by around 1% absolute per Gy EQD_2_, steeper than the rise of 0.2%/Gy found when observed survival levels for all schedules were plotted against prescribed doses (Figure 2). For cCRT, however, calculated survival levels fall with increasing dose, in line with the findings of *RTOG*-0617. Much of the additional tumoricidal effect of dose-escalation is offset by the modelled tumour repopulation and is therefore not available to counteract increases in modelled survival-limiting toxicity. For comparison, OS rates calculated for Models 1 and 2, which describe the data less well, are also plotted in the figure.

Fitted *α/β* ratios were 4.0 Gy (95% CI: 2.1, 9.2 Gy) for Model 1 and 3.0 Gy (95% CI: 1.6, 5.6 Gy) for Model 2. Both values were similar to those found in our earlier analysis of related data and were significantly lower than the generic tumour *α/β* value of 10 Gy [14], implying that for LA-NSCLC radiation cell-killing depends more strongly on the schedule dose-per-fraction than is standardly assumed. For Model 5, however, fitted stage-dependent *α/β* values were 10.0 Gy (95% CI: 0.6 Gy, infinite) for stage I/II disease, 32.1 Gy (95% CI: 9.0 Gy, infinite) for stage IIIA and 0.6 Gy (95% CI: −0.2, 1.0 Gy) for IIIB, suggesting that cell-killing depends more strongly on dose-per-fraction for stage IIIB than IIIA NSCLC.

Figure S1 shows a matrix of coefficients *r* of correlations between the fitted parameters of Model 5. The only strong correlation (|*r*| ≥ 0.7) was between *EQD*_2,*tum*50_ (*S_IIIB_*) and the repopulation rate for RT alone and sCRT. The high repopulation rate for cCRT and the low *α/β* ratio for IIIB disease, which are the novel features of the fit of Model 5, were moderately (0.3 ≤ |*r*| < 0.7) negatively correlated. The cCRT repopulation rate was also moderately correlated with a further six parameters, and the stage IIIB *α/β* ratio with a further seven. Despite these moderate parameter correlations the (−0.2, 1.0 Gy) 95% CI of the low *α/β* value for IIIB disease excluded the conventional *α*/*β* value of 10 Gy by a large margin. However, the (0.36, 2.57 Gy/day) 95% CI on the repopulation rate for cCRT did include the 0.6–0.7 Gy/day rates often used in schedule effect calculations for NSCLC [13].

Alternative model generalisations were also tested and are summarized in Table S3. Models in which tumour *α/β* varied with treatment-type rather than stage, or in which repopulation parameters varied with stage rather than treatment-type, had worse AIC and cross-validation scores than Model 5.

## 4. Discussion

2-year OS rates published for NSCLC patients have been fitted using models that included progressively more treatment- and patient-related factors, aiming to achieve a coherent description of how dose, dose-per-fraction and treatment duration might be optimised in chemoradiotherapy treatments of lung cancer. The data were described best by a model in which the fitted tumour repopulation rate varied with the use and timing of chemotherapy and fitted tumour *α/β* and toxicity parameters varied with disease stage. Modelled repopulation offset 1.47 Gy EQD_2_-per-day for cCRT, starting at day 24 of treatment, but only 0.30 Gy-per-day starting at day 17 for RT alone and sCRT. For stage IIIB disease, the tumour *α/β* ratio was lower than for other disease stages and the modelled survival-limiting toxicity rate was higher.

### 4.1. Causes and Implications of the Rapid Repopulation Rate Fitted for cCRT

The model fit suggests tumour repopulation is initially delayed by cCRT but is then fast, perhaps due to known paradoxical effects of chemotherapy which include the selection and expansion of cancer stem cell populations [24]. Recently, the lack of improved survival in a trial of moderately accelerated cCRT was tentatively attributed to suppression of repopulation by cCRT, neutralising the gains from acceleration [15]. While this concurs with the model fit’s description of a delayed start to repopulation, it is at variance with the subsequent rapid modelled acceleration. The model fit accounts for the trialled schedule’s lack of survival benefit by a different mechanism, namely the low tumour *α/β* ratio which reduces the effect of the first phase of the treatment given in 1.5 Gy fractions.

Rapid repopulation during cCRT would partly explain the poorer OS seen in the high-dose arm of *RTOG-0617*, which gave 74 Gy over a long radiation course of 51 days [12]. Relatedly, a single institution analysis of 956 patients indicated that dose-escalation only benefitted LA-NSCLC patients when cCRT was completed within 49 days [25]. Our analysis is consistent with this and suggests cCRT schedules should be limited to the shortest durations over which prescribed radiation doses can be safely delivered.

The distribution of histological subtypes differed slightly between cohorts receiving cCRT (fewer squamous cell carcinomas, more other NSCLC) and those receiving sCRT and RT alone (Table 1). While this might contribute to the difference in repopulation kinetics, a model in which repopulation depended on tumour histology did not describe the data as well as the model in which repopulation depended on treatment-type.

Although the fitted repopulation rate was 1.47 Gy EQD_2_-per-day for cCRT, this can be countered by giving only an extra 1.05 Gy-per-day of physical dose in 2 Gy fractions, due to the radiosensitising property of cCRT which is accounted for in Models 2–5 by scaling cCRT radiation doses by the *RS* factor. This 1.05 Gy-per-day figure is, however, still substantially greater than conventional estimates of 0.6–0.7 Gy lost per day to repopulation, and the associated modelled reduction in survival is high, governed either by the 1.05 Gy-per-day figure considered in relation to the radiosensitised (steepened) dose-response curve for cCRT, or equivalently by the 1.47 Gy-per-day figure in relation to the dose-response curve for sCRT. The high fitted rate of loss of cell-killing effect due to repopulation, together with increased modelled survival-limiting toxicity at higher doses, lies behind the reduction in OS calculated from the fit of Model 5 for cCRT schedules delivering greater numbers of 2 Gy fractions over longer schedules.

### 4.2. The Apparent Stage-Split in α/β

Model performance improved when *α/β* took high values for stages I-II and IIIA disease but a low value for IIIB. For early-stage NSCLC, previous studies have reported both low and high *α/β* values [26,27,28] and our findings add little information since we intentionally excluded cohorts with many stage I/II patients, and so the fitted *α/β* ratio for these patients had a broad 95% CI covering all values above 0.6 Gy. On the other hand, there was a clear split between the 32.1 Gy (95% CI: 9.0 Gy, infinite) *α/β* value we obtained for IIIA disease and the 0.6 Gy (95% CI: −0.2, 1.0 Gy) value obtained for IIIB.

**Figure 5:**
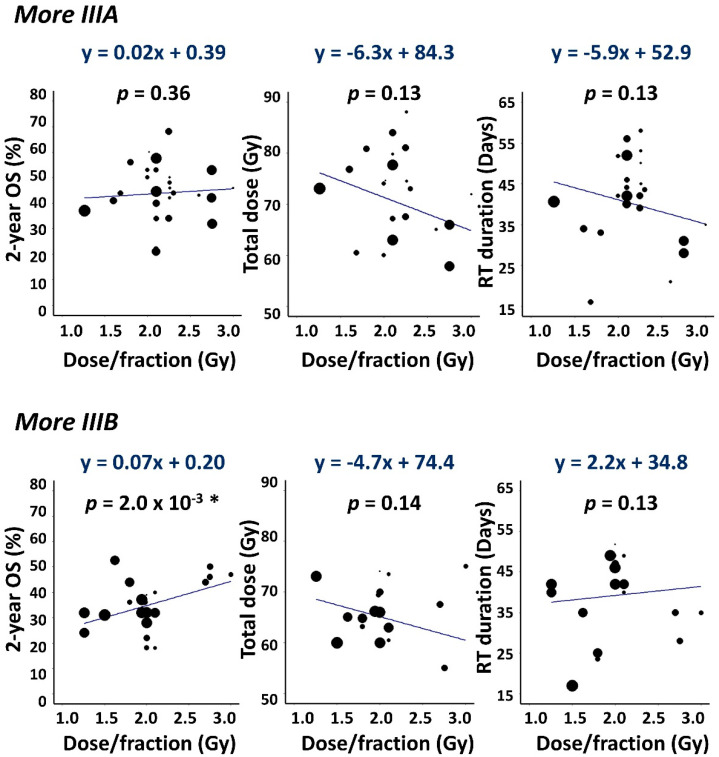
Scatter plots showing 2-year OS, total dose and RT duration versus dose-per-fraction. Data are plotted separately for *More IIIA* (IIIB/IIIA patient ratio < 1) and *More IIIB* (IIIB/IIIA > 1) cohorts together with weighted least squares linear fits to the data. Gradients with *p <* 0.05 are asterisked.

Data features underlying this *α/β* stage-split are shown in Figure 5 which plots 2-year OS, prescribed dose and RT duration against dose-per-fraction. The data are plotted separately for 27 ‘*More IIIA*’ and 22 ‘*More IIIB*’ cohorts in which IIIA/IIIB ratios of patients were respectively greater or less than 1, the remaining two cohorts having an equal balance. Survival increased notably and significantly with dose-per-fraction in *More IIIB* but not *More IIIA* cohorts, suggesting a lower *α/β* value (greater dependence of radiation cell-killing on dose-per-fraction) for IIIB disease.

We refitted the data using a version of Model 5 in which stage III patients belonging to the *More IIIA* and *More IIIB* cohorts were assigned separate *α*/*β* values. Fitted *α*/*β* ratios obtained were 12.7 Gy (95% CI: 8.4, 24.8 Gy) for the 2050 stage III patients (64% IIIA) in the *More IIIA* cohorts, and 1.2 Gy (95% CI: −0.5, 4.7 Gy) for the 2256 stage III patients (60% IIIB) in the *More IIIB* cohorts. These values differ significantly but less sharply than those obtained for IIIA and IIIB patients separately and are arguably linked more directly to the underlying data since most cohorts included both IIIA and IIIB patients.

Tumour histology seems unlikely to account for the *α*/*β* difference as the distribution of tumour subtypes was similar for stage III patients in the *More IIIA* and *More IIIB* cohorts: 42%, 30% and 28% of the *More IIIA* patients had squamous cell carcinoma, adenocarcinoma and other NSCLC histologies, respectively, compared to 46%, 28% and 26% for *More IIIB,* respectively. Moreover, a model in which *α*/*β* varied with tumour histology described the data less well than the one in which *α*/*β* varied with stage.

If *α*/*β* is truly low for NSCLC, at least for stage IIIB, then hypofractionated treatments delivering larger doses-per-fraction should perform as well as or better than conventionally fractionated treatments, particularly given that they can be delivered straightforwardly in shorter overall times. In combined immune-radiation treatments, schedules delivering higher doses-per-fraction in fewer fractions appear to be more effective, including evidence of reduced lymphopenia [29].

### 4.3. Fitted Survival-Limiting Toxicity Was Greater for Higher Stage Patients

Model performance improved when the fitted survival-limiting toxicity rate was allowed to increase with disease stage (Table S2). This was anticipated since high grade complications, or reduced treatment effectiveness due to radiation-induced immune-suppression [30,31,32], would be expected more often in higher stage patients with larger irradiated normal tissue volumes.

### 4.4. Statistical Uncertainties

This study aimed to coherently account for the effects on survival of factors including disease stage, prescribed dose, dose-per-fraction, RT duration, use and timing of chemotherapy, and possible survival-limiting toxicities. Using AIC and cross-validation techniques we chose the model that best balanced parsimony with quality of fit to the large dataset. Nevertheless, this model included 21 fitted parameters some of which were correlated, broadening 95% CI on fitted values. We therefore checked the robustness of our main findings.

Survival-limiting toxicities at high dose-levels, together with rapid modelled tumour repopulation, caused 2-year OS calculated for cCRT from the model fit to fall by 4% and 10% absolute respectively for stage IIIA and IIIB patients as prescribed dose increased from 60 to 74 Gy given in 40 and 51 days (Figure 4). Uncertainties on these figures were assessed by creating 1000 bootstrap datasets in which cohorts were sampled from the original set with replacement, refitting Model 5 to each bootstrap and using each model fit to recalculate the expected change in survival with increased dose. Average reductions in modelled survival and 95% ranges were 0% (−24, 32%) for IIIA and 6% (−16, 38%) for IIIB patients treated using cCRT, compared to increases of 23% (−18, 49%) and 16% (−23, 39%) for sCRT. In 96% of the bootstrap resamples, changes in survival with dose were lower (less of an increase/more of a reduction) for cCRT than for sCRT treatment. In 93% of bootstraps the fitted repopulation rate was faster for cCRT. Results for RT alone were similar to those for sCRT.

In summary, the model fit to the original dataset indicated a survival reduction with increased dose given in 2 Gy fractions, as did average results from model fits to the bootstraps. In most bootstraps the fall in survival was greater for cCRT than for sCRT, and the fitted rate of repopulation was faster for cCRT. Thus, it can robustly be concluded that dose-escalation with treatment protraction is a poorer option for cCRT than for sCRT or RT alone.

The *α*/*β* results were also statistically robust. The overall *α*/*β* ratios of 4.0 and 3.0 Gy fitted for Models 1 and 2 were significantly lower than the conventional value of 10 Gy. The fitted *α*/*β* values of 12.7 and 1.2 Gy for stage III patients in the *More IIIA* and *More IIIB* cohorts differed significantly, and the same was true of *α*/*β* values obtained separately for IIIA and IIIB patients in the fit of Model 5.

As with many datasets compiled from multiple sources, even for the best-performing model the scale of differences between the model fits and observed OS rates was higher than expected on the basis of binomial statistical uncertainties alone [33,34,35]. This ‘overdispersion’ phenomenon results from centre-to-centre differences in patient and treatment factors that are not accounted for in the modelling, generally because data for them is unavailable. For the fit of Model 5, the root mean square difference between modelled and observed OS rates was 34% greater than expected. Overdispersion is often ignored but can be worked into the modelling process by dividing goodness-of-fit measures by an overdispersion factor. The effect is to reduce the measures to levels expected from binomial statistics, widen CI on fitted parameters, and potentially cause simpler models to be preferred over more complex ones, since in the AIC the penalty term for increased numbers of model factors remains unchanged whereas the goodness-of-fit factor is weighted down. Factoring overdispersion into the analysis presented here leads to Model 4 having a marginally better AIC than Model 5 and thus being marginally preferred if overdispersion is accounted for. The fit of Model 4 with CI widened to account for overdispersion is shown in Table S4. As can be seen, even allowing for overdispersion the analysis continues to point to a low *α*/*β* ratio for stage IIIB NSCLC but a significantly higher *α*/*β* ratio for stage IIIA, and to the repopulation rate during cCRT being significantly faster than during sCRT or RT alone.

### 4.5. Study Limitations

Aside from the factors studied in this retrospective analysis, inevitable cohort-to-cohort variations in other patient- and treatment-related characteristics may not only lead to overdispersion but also potentially bias the results obtained. The American Joint Committee on Cancer staging system evolved from version 4 to 7 between 2000 and 2016, and this may cause some stage definition inconsistency [36]. Additionally, we lacked molecular and stage-specific histological information to further refine the analysis.

Normal tissue radiation doses were not detailed in most data sources. The survival-limiting toxicity terms in Models 2–5 therefore presuppose that such toxicities arise in normal tissues lying close to the tumour and their incidence is linked to the prescribed dose-level. This approximate approach provided a better description of the data than was achieved when the toxicity term was not factored into the analysis.

### 4.6. Future Directions

To check the predictive ability of the Model 5 fit, 2-year OS was calculated for the baseline arm of the PACIFIC trial of cCRT alone versus combined immune-cCRT [37], which was not included in the fitted dataset. The 54–66 Gy prescribed doses received by 92% of PACIFIC patients were approximated to 60 Gy, the 66–74 Gy doses received by 8% of patients to 70 Gy, and the 53:45:2% split for stage IIIA/IIIB/other to 50:50:0%. The calculated OS was 56.1% for Model 5 (50.1–55.6% for Models 1–4), close to the observed level of 55.6%. This sets up the model well as a baseline for future work characterising the additional effects on survival of combined immune-chemo-RT treatments, as given in the experimental arm of PACIFIC, and their dependence on the scheduling of chemo-RT and immune drug delivery.

## 5. Conclusions

In this retrospective analysis of published data, 2-year OS was described best by a model fit in which tumour accelerated repopulation progressed at 1.47 Gy EQD_2_-per-day for cCRT compared to 0.30 Gy per day for sCRT and RT alone. These rates suggest that cCRT treatments should be given in the shortest times in which prescribed doses can be tolerated, but that treatment acceleration offers less advantage for RT alone or sCRT. Hypofractionation provides one way of accelerating cCRT, and the low overall fitted *α/β* ratio of 3.0 Gy suggests this approach should be efficacious as well as practical provided normal tissue EQD_2_s are not increased in the process. It should, though, be noted that when split by cohort-type, *α/β* was low only for cohorts with more IIIB than IIIA patients.

## Figures and Tables

**Table 1:** Numbers of patients with stages I-IIIB NSCLC in 51 cohorts grouped by treatment-type (radiotherapy (RT) alone, sequential chemoradiotherapy (sCRT) or concurrent chemoradiotherapy (cCRT)), listed with other patients and treatment characteristics.

	RT Alone	sCRT	cCRT	Total
Cohorts	10	26	15	51
Patient numbers				
Stage I	116	127	3	246 (5.1%)
Stage II	72	85	59	216 (4.4%)
Stage IIIA	514	732	1020	2266 (46.6%)
Stage IIIB	490	848	800	2138 (43.9%)
Total	1192 (24.5%)	1792 (36.8%)	1882 (38.7%)	4866 (100%)
Histology * (% of patients): squamous cell carcinoma/adenocarcinoma/other NSCLC				
	49.4/24.2/26.4	42.5/32.8/24.7	38.2/31.0/30.8	42.6/29.6/27.8
RT technique (# of cohorts): rectangular field RT/conformal RT/intensity-modulated RT ^†^				
	2/7/1	3/22/1	0/11/4	5/40/6
IIIB/IIIA patient ratio in each cohort: mean (range)				
	0.92 (0–1.92)	1.22 (0–2.89)	0.83 (0.16–1.72)	1.05 (0–2.89)
Prescribed dose ^††^ (Gy): mean (range)				
	71 (58–81)	70 (55–95)	69 (55–78)	70 (55–95)
RT fractions (#): mean (range)				
	36 (20–58)	33 (20–43)	36 (20–58)	35 (20–58)
Dose-per-fraction ^††^ (Gy): mean (range)				
	2.0 (1.3–3.0)	2.0 (1.7–2.8)	2.0 (1.3–2.8)	2.0 (1.3–3.0)
RT duration (days): mean (range)				
	35 (17–46)	42 (16–60)	43 (28–52)	41 (16–60)
OS_2-year_ (%): mean (range)				
	36 (21–56)	37 (18–59)	49 (32–68)	41 (18–68)

* Histology data were only available for 39/51 cohorts (9 RT alone, 16 sCRT, 14 cCRT). ^†^ In two IMRT cohorts some patients were treated using conformal RT. ^††^ Doses have been increased by 5% above published values for cohorts planned without lung tissue heterogeneity correction.

**Table 2:** Summary of key model fits showing degrees of freedom (df), fitted parameter values and 95% profile-likelihood confidence intervals, log-likelihood values, Akaike information criterion (AIC) and cross-validation scores.

Parameters	Model 1	Model 2	Model 5
	(df = 43)	(df = 38)	(df = 30)
*λ* * (Gy/day)	0.64 (0.30–0.99)	0.40 (0.23–0.72)	*RT & sCRT*: 0.30 (0.18–0.47)
			*cCRT*: 1.47 (0.36–2.57)
*T_k_* * (days)	33 (18–39)	25 (16 ^†^–36)	*RT & sCRT*: 17 (16 ^†^–32)
			*cCRT*: 24 (16 ^†^–47)
*α/β* * (Gy)	4.0 (2.1–9.2)	3.0 (1.6–5.6)	*S_I_ & S_II_*: 10.0 (0.6–infinite)
			*S_IIIA_*: 32.1 (9.0–infinite)
			*S_IIIB_*: 0.6 (−0.2–1.0)
*EQD*_2*,tum*50_ (Gy) ^††^	*S_I_*: 74 (66–81)	48 (13–53)	54 (49–58)
	*S_II_*: 74 (66–84)	48 (19–52)	54 (49–58)
	*S_IIIA_*: 74 (66–84)	49 (42–54)	54 (49–58)
	*S_IIIB_*: 88 (75–103)	61 (52–69)	54 (49–58)
*m*	0.72 (0.64–1.00 ^†^)	0.28 (0.19–0.47)	0.15 (0.12–0.24)
*OS_max_*(*CRT*) (%)	-	93 (85 ^†^–100 ^†^)	91 (85 ^†^–100 ^†^)
*RS*(*cCRT*)	-	1.11 (1.05–1.22)	1.40 (0.99–1.40 ^†^)
*EQD*_2*,NT*50_ (Gy)	-	96 (83–116)	54 (26–104)
*m_NT_*	-	0.60 (0.45–1.00^†^)	0.31 (0.11–1 ^†^)
*R* (per year)	-	0.016 (0.006–0.022)	0.016 (0.012–0.026)
*F_SLT_* ^††^	-	-	*S_I_*: 0.33 (0 ^†^–0.78)
			*S_II_*: 0.33 (0 ^†^–0.86)
			*S_IIIA_*: 0.41 (0.20–1 ^†^)
			*S_IIIB_*: 0.58 (0.35–1 ^†^)
−2 log-likelihood	6468.6	6364.8	6329.4
		(*p* < 10^−20^ cf Model 1)	(*p* < 10^−22^ and 10^−4^
			cf Models 1 and 2)
AIC score	6485	6391	6371
Cross-validation	65.2	26.7	12.7
score			

* In Model 5, fitted *λ* and *T_k_* values were allowed to differ for cCRT versus RT alone and sCRT treatments, and fitted *α/β* values to differ between stages I & II (*S_I_* & *S_II_*), stage IIIA and stage IIIB NSCLC. ^†^ The profile-likelihood confidence interval was truncated at the lower or upper boundary of the range explored. ^††^ Fitted values of *EQD*_2*,tum*50_ and *F_SLT_* were constrained so that stage IIIB values were ≥ IIIA ≥ II ≥ I. Parameters: *λ*, dose-per-day repopulated; *T_k_*, repopulation kick-off time; *α/β*, tumour fractionation dependence; *EQD*_2*,tum*50_, EQD_2_ required to achieve 50% tumour control; *m*, tumour dose-response relative gradient; *OS_max_*(*CRT*), maximum overall survival for chemoradiotherapy; *RS*(*cCRT*), radiosensitisation of dose-effects by cCRT; *EQD_2,NT50_*, EQD_2_ causing a 50% modelled survival-limiting toxicity rate; *m_NT_*, survival-limiting toxicity response relative gradient; *R*, variation of 2-year OS with study publication year; *F_SLT_*, survival-limiting toxicity weighting for stage *S_i_*.

## Data Availability

Research data are stored in an institutional repository and will be shared upon request to the corresponding author.

## Supplementary Materials

The following supporting information can be downloaded at: https://www.mdpi.com/article/10.3390/cancers14194869/s1, Table S1: Fits of all models, showing fit degrees of freedom (df), fitted parameter values and 95% profile-likelihood CI, log-likelihood values and Akaike information criterion (AIC) scores; Table S2: Comparison of the qualities of fits of the five models, showing degrees of freedom (df), log-likelihoods, likelihood-ratio tests, AIC scores and 10-fold cross-validation; Table S3: Fit of Model 5 compared to fits of (A) alternative models in which parameters were individualised as specified in the footnotes, (B) models in which some parameters that varied with treatment or stage in Model 5 were set to single common values as indicated; Table S4: AIC scores for Models 1–5 adjusted for overdispersion, and the fit of Model 4 with CI also adjusted for overdispersion; Figure S1: Correlation matrix for the fitted parameter values of Model 5. The heat scale of correlation coefficients *r* is shown on the right.
