# Supplementary material for: Dose-Response Analysis Describes Particularly Rapid Repopulation of Non-Small Cell Lung Cancer during Concurrent Chemoradiotherapy
Source: Cancers (Basel). 2022 Oct 5;14(19):4869. doi: 10.3390/cancers14194869 (PMC9563948; doi:10.3390/cancers14194869)

Supplementary Materials

Table S1. Fits of all models, showing fit degrees of freedom (df), fitted parameter values and 95% profile-likelihood CI, log-likelihood values and Akaike Information Criterion (AIC) scores.

Parameters	Model 1 (df = 43)	Model 2 (df = 38)	Model 3 (df = 36)	Model 4 (df = 34)	Model 5 (df = 30)
λ (Gy/day)	0.64 (0.30 - 0.99)	0.40 (0.23 - 0.72)	*RT/sCRT*: 0.32 (0.20 - 0.49)<br>*cCRT*: 1.00 (0.47 - 1.74)	0.33 (0.22 - 0.60)<br>1.31 (0.78 - 2.43)	0.30 (0.18 - 0.47)<br>1.47 (0.36 - 2.57)
T_k (days)	33 (18 - 39)	25 (16† - 36)	*RT/sCRT*: 16 (16† - 30)<br>*cCRT*:35 (16† - 42)	16 (16† - 25)<br>38 (16† - 46)	17 (16† - 32)<br>24 (16† - 47)
α/β* (Gy)	4.0 (2.1 - 9.2)	3.0 (1.6 - 5.6)	2.6 (1.5 - 4.5)	S_I & S_{II}: 10.2 (0.6 - infinite)<br>S_{IIIA}: 62.8 (17.1 - infinite)<br>S_{IIIB}: 0.4 (-0.3 - 1.0)	10.0 (0.6 - infinite)<br>32.1 (9.0 - infinite)<br>0.6 (-0.2 - 1.0)
$EQD_{2,tum50}$ (Gy) ††	S_I: 74 (66 - 81)<br>S_{II}: 74 (66 - 84)	48 (13 - 52)<br>48 (19 - 52)	49 (13 - 53)<br>49 (13 - 54)	54 (13 - 56)<br>54 (19 - 57)	54 (49 - 58)<br>54 (49 - 58)

	S_{IIIA}: 74 (66 - 84)	49 (42 - 54)	49 (44 - 54)	54 (50 - 57)	54 (49 - 58)
	S_{IIIB}: 88 (75 - 103)	61 (52 - 69)	58 (53 - 67)	54 (50 - 57)	54 (49 - 58)
m	0.72 (0.64 - 1.00†)	0.28 (0.19 - 0.47)	0.25 (0.17 - 0.43)	0.16 (0.13 - 0.26)	0.15 (0.12 - 0.24)
$OS_{max}(CRT)$ (%)	-	93 (85† - 100†)	91 (85† - 100†)	85 (85† - 98)	91 (85† - 100†)
RS^{cCRT}	-	1.11 (1.05 - 1.22)	1.10 (0.99 - 1.40†)	1.10 (0.99 - 1.40†)	1.40 (0.99 - 1.40†)
$EQD_{2,NT50}$ (Gy)	-	96 (83 - 116)	96 (85 - 114)	96 (85 - 111)	54 (26 - 104)
m_{NT}	-	0.60 (0.45 - 1.00†)	0.60 (0.45 - 1.00†)	0.60 (0.42 - 1.00†)	0.31 (0.11 - 1.00†)
R (per year)	-	0.016 (0.006 - 0.022)	0.016 (0.008 - 0.023)	0.015 (0.009 - 0.022)	0.016 (0.012 - 0.026)
F_{SLT} ††	-	-	-	-	S_I: 0.33 (0† - 0.78)<br>S_{II}: 0.33 (0† - 0.86)<br>S_{IIIA}: 0.41 (0.20 - 1†)<br>S_{IIIB}: 0.58 (0.35 - 1†)
-2log-likelihood	6468.6	6364.8	6356.7	6339.2	6329.4
AIC score	6485	6391	6387	6373	6371

* In Model 5, λ and T_k were set to common values for RT alone and sCRT treatments, and α/β was set to a common value for stages I and II (S_I and S_{II}) NSCLC.

† The profile-likelihood confidence interval was truncated at the lower or upper boundary of the range explored.

†† Fitted values of $EQD_{2,tum50}$ and F_{SLT} were constrained so that stage IIIB values were ≥ IIIA ≥ II ≥ I.

Parameters: λ, dose-per-day repopulated; T_k, repopulation kick-off time; α/β, tumour fractionation dependence; $EQD_{2,tum50}$, EQD_2 required to achieve 50% tumour control; m, tumour dose-response relative gradient; $OS_{max}(CRT)$, maximum overall survival for chemoradiotherapy; $RS(cCRT)$, radiosensitization of dose-effects by cCRT; R, variation of 2-year OS with study publication year; $EQD_{2,NT50}$, EQD_2 causing a 50% modelled survival-limiting toxicity rate; m_{NT}, survival-limiting toxicity response relative gradient; F_{SLT}, survival-limiting toxicity weighting for stage S_i.

Table S2. Comparison of the qualities of fits of the five models, showing degrees of freedom (df), log-likelihoods, likelihood-ratio tests, AIC scores and 10-fold cross-validation.

	Model 1 (df = 43)	Model 2 (df = 38)	Model 3 (df = 36)	Model 4 (df = 34)	Model 5 (df = 30)
-2 log-likelihood	6468.6	6364.8	6356.7	6339.2	6329.4
Likelihood-ratio test	Reference	$p < 10^{-20}$	$p < 10^{-20}$	$p < 10^{-22}$	$p < 10^{-22}$
	-	Reference	$p = 0.02$	$p < 10^{-4}$	$p < 10^{-4}$
	-	-	Reference	$p < 10^{-3}$	$p < 10^{-3}$
	-	-	-	Reference	$p = 0.03$
AIC score	6485	6391	6387	6373	6371
Cross-validation score	65.2	26.7	24.1	18.0	12.7

Table S3. Fit of Model 5 compared to fits of (A) alternative models in which parameters were individualised as specified in the footnotes, (B) models in which some parameters that varied with treatment or stage in Model 5 were set to single common values as indicated.

(A)

	Model 5 (df = 30)	Model 5-1 (df = 31)	Model 5-2 (df = 28)	Model 5-3 (df = 32)	Model 5-4 (df = 28)
-2 log-likelihood	6329.4	6333.4	6333.8	6332.2	6326.6
AIC score	6371	6373	6380	6370	6373

Model 5-1 α/β individualised by treatment (cCRT vs. others) rather than stage
Model 5-2 λ and T_k individualised by stage (early, IIIA, IIIB) rather than treatment
Model 5-3 Toxicity weighting (F) individualised by treatment (cCRT vs. others) rather than stage
Model 5-4 λ and T_k individualised by RT, sCRT, cCRT rather than cCRT vs. others

(B)

	Model 5 (df = 30)	Model 5 with common α/β for all stages (df = 32)	Model 5 with common repopulation for all treatments (df = 32)	Model 5 with common toxicity for all stages (df = 34)
-2 log-likelihood	6329.4	6343.8	6335.6	6339.8
AIC score	6371	6382	6374	6374

Table S4. AIC scores for Models 1-5 adjusted for overdispersion, and the fit of Model 4 with CI also adjusted for overdispersion.

	Model 1	Model 2	Model 3	Model 4	Model 5
AIC considering overdispersion	3610	3562	3561	3556	3558
Likelihood-ratio test	Reference	$p < 10^{-10}$	$p < 10^{-10}$	$p < 10^{-11}$	$p < 10^{-10}$
	-	Reference	$p = 0.12$	$p = 0.01$	$p = 0.01$
	-	-	Reference	$p = 0.01$	$p = 0.01$
	-	-	-	Reference	$p = 0.22$

Parameters	Model 4 with overdispersion correction
λ (Gy/day)	*RT/sCRT*: 0.33 (0.20 - 0.63)<br>*cCRT*: 1.19 (0.66 - 2.41)
T_k (days)	*RT/sCRT*: 16 (16† - 28)<br>*cCRT*: 38 (16† - 46)
α/β (Gy)	$S_{I/II}$: 10.2 (0.4 - infinite)<br>S_{IIIA}: 62.8 (14.6 - infinite)<br>S_{IIIB}: 0.4 (-0.4 - 1.2)
$EQD_{2,tum50}$ (Gy)	S_{I}: 54 (12 - 57)<br>S_{II}: 54 (18 - 57)<br>S_{IIIA}: 54 (50 - 57)<br>S_{IIIB}: 54 (48 - 59)
m	0.16 (0.12 – 0.27)
$OS_{max}(CRT)$ (%)	85 (85† - 98)
RS^{cCRT}	1.10 (0.97 - 1.40†)

R (per year)	0.015 (0.007 - 0.023)
$EQD_{2,NT50}$ (Gy)	96 (84 - 113)
m_{NT}	0.60 (0.40 - 1.00)

Figure S1. Correlation matrix for the fitted parameter values of Model 5. The heat scale of correlation coefficients r is shown on the right.

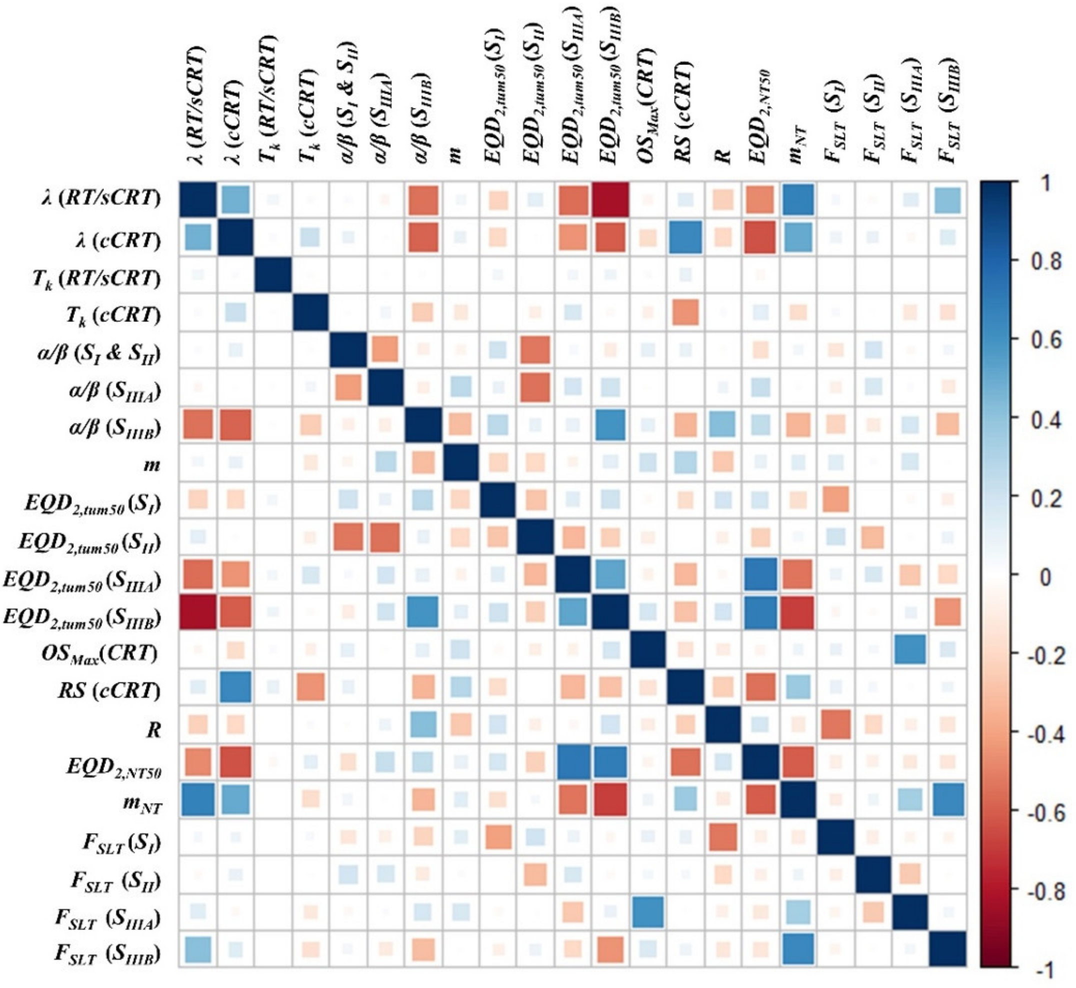

Supplement: Supplementary file 1 [file cancers-14-04869-s001.zip › cancers-1908030-supplementary.pdf]
